# Candida glabrata and the host: a neglected affair

**DOI:** 10.1099/jmm.0.002161

**Published:** 2026-05-08

**Authors:** Manisha Ghosh, Mayur Raney, Rupinder Kaur

**Affiliations:** 1 BRIC-Centre for DNA Fingerprinting and Diagnostics (CDFD), Hyderabad, 500039, Telangana, India; 2 Graduate Studies, Regional Centre for Biotechnology, Faridabad, 121001, Haryana, India; 3 Graduate Studies, Manipal Academy of Higher Education, Manipal, 576104, Karnataka, India

**Keywords:** chromatin architecture, genome evolution, host–pathogen dynamics, human fungal pathogens, innate immune system, metabolism reprogramming

## Abstract

*Candida glabrata* (*Nakaseomyces glabratus*), a prevalent opportunistic pathogenic yeast of humans, has been classified as a fungal pathogen of high priority by the World Health Organization. *C. glabrata* is the leading cause of invasive *Candida* infections in hospitalized and immunocompromised patients and displays co-resistance to azole and echinocandin drugs. *C. glabrata* is known for its distinct virulence attributes that facilitate infections of the host in the absence of classical virulence factors, viz. filament formation and secreted toxin. The inability of *C. glabrata* to induce a strong inflammatory response contributes to its persistence in the host. However, the molecular underpinnings that define the *C. glabrata*–host relationship are not well-understood. In this review, we summarize the findings on the interplay between *C. glabrata* and different host cell types and outline the molecular strategies that play a pivotal role in the establishment of *C. glabrata* infections. Our goal is to expand our knowledge of *C. glabrata*–host crosstalk and underscore the vital questions whose resolution is essential to effectively manage *C. glabrata* infections.

## Introduction

*Candida glabrata* (*Nakaseomyces glabratus*), an asexual haploid budding yeast, is a commensal of mucosal surfaces in humans [1,3]. Although phylogenetically and morphologically distinct from the prevalent pathogenic species of the *Candida* genus, *C. glabrata* causes a similar spectrum of diseases including oral candidiasis, vulvovaginal candidiasis, candiduria and candidaemia [1,4]. *C. glabrata* prevalence varies in different geographical regions, with it being the second most common causative agent of bloodstream infections after *Candida albicans* in the USA, northern Europe, the Republic of Korea and Canada [5,9]. *C. glabrata* ranks high amongst invasive infection-causing *non-albicans Candida* species in many countries including Spain, Brazil, Japan, Australia, India and Pakistan [5,7, 9,12]. *C. glabrata* infections are frequent in Intensive Care Unit patients on prolonged antibiotic therapy and diabetic and elderly patients with a weak immune system [1,3]. The 30-day mortality rate associated with *C. glabrata* bloodstream infections ranges from 19 to 49% [7].

*C. glabrata* is also intrinsically less susceptible to the azole class of antifungals that inhibit biosynthesis of the major sterol, ergosterol, of the plasma membrane [13,14]. Further, co-resistance to azoles and the cell wall-targeting echinocandin drugs is increasingly being reported in *C. glabrata* isolates in hospitals worldwide [2,3, 7], thereby rendering *C. glabrata* infections difficult to treat. *C. glabrata* also exhibits high resistance to low pH, thermal and oxidative stresses which may contribute to its fitness in the host [2,4].

*C. glabrata* inhabits the oral cavity, genitourinary tract and gastrointestinal tract in the healthy human host, is not known to switch to the invasive hyphal form and exists primarily solely in the yeast form, although pseudohyphal structures have been reported under few environmental conditions [1,4]. *C. glabrata* does not belong to the class of aggressive pathogens and neither possesses secreted proteolytic activity nor secretes toxins [2,4]. It is widely believed that *C. glabrata* predominantly employs stealth and evasion-based strategies to persist and cause infections in the host, and its persistence is normally not associated with severe inflammation and/or host damage [2,4]. The key *C. glabrata*- and *C. albicans*–host interaction mechanisms are listed in Table 1.

**Table 1.** A comparative summary of *C. glabrata–* and *C. albicans*–host cell interaction

Trait	*C. glabrata*	*C. albicans*
Morphology	Yeast only	Yeast, pseudohyphae and hyphae
Invasion of host epithelial cells	Minimal	Substantial
Survival of infected host macrophages	High	Low
Cytokine induction	Weak	Strong
Host tissue damage	Low	High
Immune evasion	Prominent	Moderate
Infection strategy	Stealth (Trojan horse)	Aggressive

Despite *C. glabrata*–host interplay dictating the final outcome of *C. glabrata* infections, our understanding of how *C. glabrata* and host cells respond to each other during confrontation in varied host niches remains poor. In the current article, we discuss the major molecular players that mediate the crosstalk between *C. glabrata* and host cells ranging from *C. glabrata* protease and adhesin proteins, host cell receptors, immune responses to *C. glabrata* killing by neutrophils. Additionally, how chromatin and metabolic reprogramming facilitate *C. glabrata* survival in diverse host niches is discussed.

### Innate immune system

**Fig. 1.**
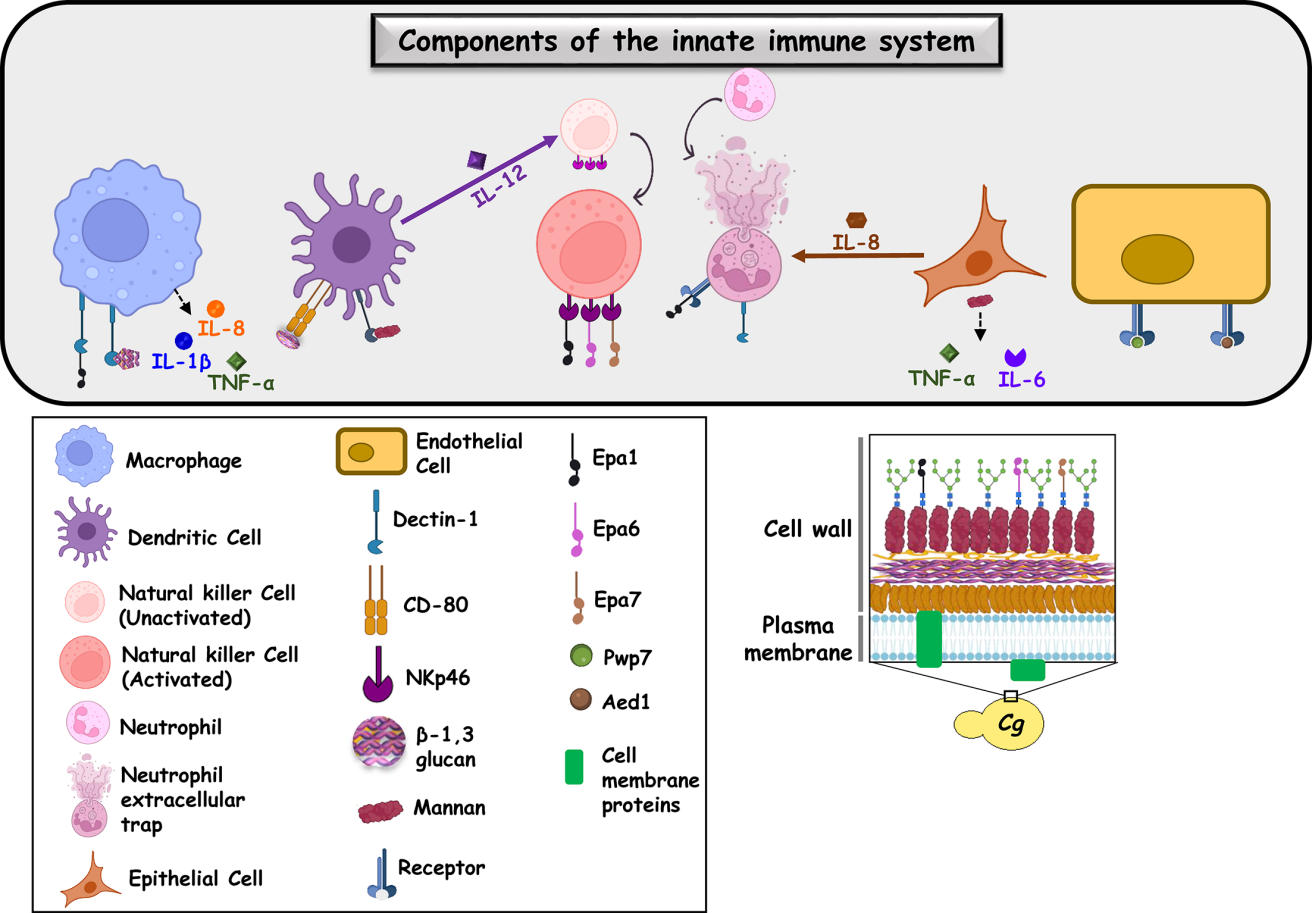
A schematic illustrating the crosstalk between *C. glabrata* and constituents of the host innate immune system. The varied pathogen-associated molecular patterns on *C. glabrata* cell surface are also shown pictorially. Dendritic cell-secreted IL-12 aids in the activation of natural killer cells, while diminished IL-8 secretion by epithelial cells impedes neutrophil recruitment.

The innate immune system, consisting of mechanical, cellular and chemical entities, serves as the first barrier against microbial infections and is pivotal to microbial pathogen recognition, appropriate signalling pathway activation and mounting of an optimal anti-microbial response to restrain, kill and eradicate the infectious pathogen [15,16]. Cellular components of the mammalian innate immune system include phagocytic, antigen-presenting and epithelial cells [15,16]. Macrophages, neutrophils, dendritic cells and natural killer cells have been implicated in immune defence against *Candida* pathogens [17]. Fig. 1 schematically illustrates the crosstalk between *C. glabrata* and various components of the innate immune system. Notably, while the interplay between *C. glabrata* and macrophages has been extensively studied, *C. glabrata*–neutrophil/natural killer/dendritic/epithelial cell interactions are beginning to be defined, as briefly summarized below:

### *C. glabrata-*macrophage dynamics

Macrophages are the key phagocytic cells of the innate immune system and pivotal to managing *C. glabrata* infections [4,17]. *C. glabrata* is known to be phagocytosed by murine and human cultured macrophage cells as well as by primary mouse peritoneal macrophages and human peripheral blood monocyte-derived macrophages [3,18,21]. It inhibits acidification of the phagolysosome and proliferates modestly in a modified (nonacidic) phagosome organelle, without significantly impacting the host macrophage physiology [19,21]. The catalytic (CgVps34) and regulatory (CgVps15) subunits of the sole phosphoinositide 3-kinase (produces phosphatidylinositol 3-phosphate) and *α*-1,6-mannosyltransferases Mnn10 and Mnn11 have been shown to be important for impeding phagosome maturation [20,22]. Although the capability of *C. glabrata* to alkalinize the external environment via amino acid utilization as carbon sources *in vitro* may contribute to the inhibition of phagolysosome acidification in host macrophages, the precise underlying molecular mechanism/s remain undefined [20]. Importantly, both phosphoinositide 3-kinase and eleven glycosylphosphatidylinositol-anchored aspartyl proteases (CgYapsins, CgYps1-11) are required for *C. glabrata* survival in macrophages, as deletion of *CgVPS15/CgVPS34* or *CgYPS1-11* genes led to intracellular killing of *C. glabrata* cells [18,21], though CgYapsins do not play a role in inhibiting acidification of the phagolysosome in *C. glabrata*-infected macrophages [21]. Notably, CgYapsins 3 and 9 have recently been postulated to act as immune-activating factors in larvae protection in the *Galleria mellonella* infection model [23]. Further, the simultaneous loss of CgSod1 superoxide dismutase and CgYap1 transcription factor that are implicated in superoxide detoxification, rendered *C. glabrata* cells inviable in macrophages [24], thereby underscoring the importance of neutralization of host-generated reactive oxygen species for intracellular survival of *C. glabrata*.

Importantly, the loss of genes implicated in iron homeostasis, pexophagy, MAPK signalling, cell wall remodelling, chromatin reorganization, DNA damage repair, biotin uptake, calcium signalling and TOR signalling results in diminished intracellular replication of *C. glabrata*, highlighting that *C. glabrata* proliferation in macrophages is probably dependent upon multiple factors that drive nutrient acquisition and/or varied stress survival mechanisms [4,25,27]. Further, it has been reported that CgYps1-11 proteases are pivotal to suppress spleen tyrosine kinase (Syk) and NLRP3 inflammasome activation and restrain proinflammatory cytokine IL-1*β* secretion in macrophages [28]. *C. glabrata* infection does not invoke substantial cytokine production, as only a small amount of GM-CSF, IL-8, IL-10 and TNF-*α* secretion was found in *C. glabrata*-infected macrophages [19,29]. Consistent with this, *C. glabrata* infection also did not lead to appreciable phosphorylation of Erk1/2 (extracellular signal-related kinases), p38 MAPK or SAPK/JNK (stress-activated protein kinases/c-jun amino-terminal kinases) [20,29]. Furthermore, a recent study has implicated CgKsp1 kinase in modulating the macrophage cytokine response, with prolongation and generation of the intramacrophage phase and stress-resistant variants, respectively, promoting *C. glabrata* survival in host macrophages [30].

Recently, the SWI/SNF chromatin remodelling complex-mediated nucleosome dynamics in macrophage-internalized *C. glabrata* cells has been implicated in suppressing the macrophage proinflammatory response by downregulating and upregulating the gene expression of immunostimulatory cell surface Epa1 adhesin and immunosuppressive mannosyltransferases, respectively [31]. It was observed that *EPA1* expression is controlled by the SWI/SNF chromatin remodelling complex-mediated nucleosome dynamics, with *CgSNF2* (ATPase subunit of the SWI/SNF complex) and *EPA1* deletion increasing and decreasing the secretion of the proinflammatory cytokine IL-1*β*, respectively, in human macrophages [31]. Of note, Epa1 adhesin is also recognized by the NKp46 receptor in natural killer cells, which leads to *C. glabrata* clearance [32], and its expression in the non-pathogenic yeast *Saccharomyces cerevisiae* promoted yeast phagocytosis and TNF-*α* and IL-8 cytokine secretion in human peripheral blood mononuclear cell-derived macrophages [33]. Further, other members of the *C. glabrata* Epa protein family including Epa6 and Epa7, along with other adhesin proteins such as Awp2, Awp3, Awp5 and Awp6 have been implicated in biofilm formation on different surfaces [34,35].

The transcriptional responses of *C. glabrata* towards the macrophage internal milieu and of the macrophages to *C. glabrata* ingestion have also been studied. *C. glabrata* activates genes belonging to the glyoxylate cycle, *β*-oxidation of fatty acids, gluconeogenesis, amino acid biosynthesis, methylcitrate cycle, TCA cycle, iron homeostasis, sterol uptake and ammonium transport inside macrophages [4,18, 25, 31, 36]. These results suggest that *C. glabrata* may utilize glyoxylate shunt to utilize two-carbon compounds in macrophages. Further, genes implicated in translation, ergosterol biosynthesis, high-affinity iron uptake and cell wall metabolism were repressed in macrophage-internalized *C. glabrata* cells [18,25]. Of note, the transcriptional factor CgXbp1, via elongating RNA polymerase-II ChIP analysis, was found to be a key player in shaping the temporal transcriptional landscape of macrophage-internalized *C. glabrata* cells [36]. Intriguingly, *C. glabrata* infection to human macrophages led to an increased expression of the genes belonging to many biological processes including chemotaxis, negative regulation of inflammatory response, response to hypoxia, ion transport and positive regulation of cytosolic calcium ion concentration in macrophages [28]. Contrarily, human macrophages exhibited a reduced expression of the genes implicated in tumour necrosis factor-mediated signalling, positive regulation of MAPK cascade, apoptotic signalling pathway and positive regulation of phosphatidylinositol 3-kinase signalling pathways, in response to *C. glabrata* infection [28]. These transcriptional profiles suggest that differential expression of the genes implicated in inflammatory response, chemotaxis, MAPK signalling pathway and apoptosis largely constitute the macrophages’ response to *C. glabrata* ingestion [28].

The *C. glabrata* cell wall constituents, *β*-1,3 glucan, mannan and mannoproteins, may act as major pathogen-associated molecular patterns that are recognized by host cell receptors, with the inner *β*-1,3 glucan core usually concealed by the outer mannan layer [37,39]. For *C. glabrata* uptake, the C-type lectin receptor, Dectin-2 (binds to *α*-mannan and *β*-glucan), has been implicated, as macrophages of dectin-2^−/−^ (Dectin-2-knockout) mice were deficient in *C. glabrata* phagocytosis [40]. Importantly, *C. glabrata* displayed similar binding to the wild-type and Dectin-1^−/−^ (Dectin-1-knockout) immortalized murine macrophages, suggesting that *C. glabrata* binding to macrophages may be independent of the *β*-glucan receptor, Dectin-1 [33]. However, macrophages of Dectin-1^−/−^ mice mounted a low inflammatory response, with Dectin-1^−/−^ mice also showing impaired immune response development to *C. glabrata*, thereby implicating Dectin-1 receptor in controlling *C. glabrata* infections [29]. The major immune evasion and persistence strategies of *C. glabrata* are listed in Table 2.

**Table 2.** Immune evasion and persistence strategies of *C. glabrata*

Strategy	Mechanism	Outcome	Key references
Masking of the cell wall *β*-glucan	Mannan/mannoprotein-rich cell wall	Avoid recognition by PRR	[6,22, 29, 37,40]
Intracellular survival	Blocking phagosome maturation ROS detoxification Metabolic reprogramming	Avoid killing and promote proliferation in macrophages	[19,21,24, 26, 36]
Cytokine suppression	Dampened macrophage signalling, Dampened epithelial cell signalling	Survival in macrophages and diminished neutrophil recruitment	[19,28, 43, 53]
Chromatin reorganization-based gene expression regulation of immunomodulatory molecules	Variable cell wall molecule expression	Avoid recognition by host immune cell receptors	[31,114, 115, 118]
Dynamic genome and gene regulation mechanisms	Maintenance of genome integrity and maximal nutrient acquisition inside the host	Facilitate survival and replication	[25,27,31, 36, 69, 70, 108, 110, 112, 114, 115]
Acquisition of drug resistance	Activation of drug efflux, modification of drug target	Limit drug efficacy	[41,113, 123 and reviewed in [7,13, 14]
Interaction with host proteins and *C. albicans*	Increased invasion potential	Gain entry into host tissue	[32,33, 53, 60,64, 66, 67, 76, 77]

**Fig. 2.**
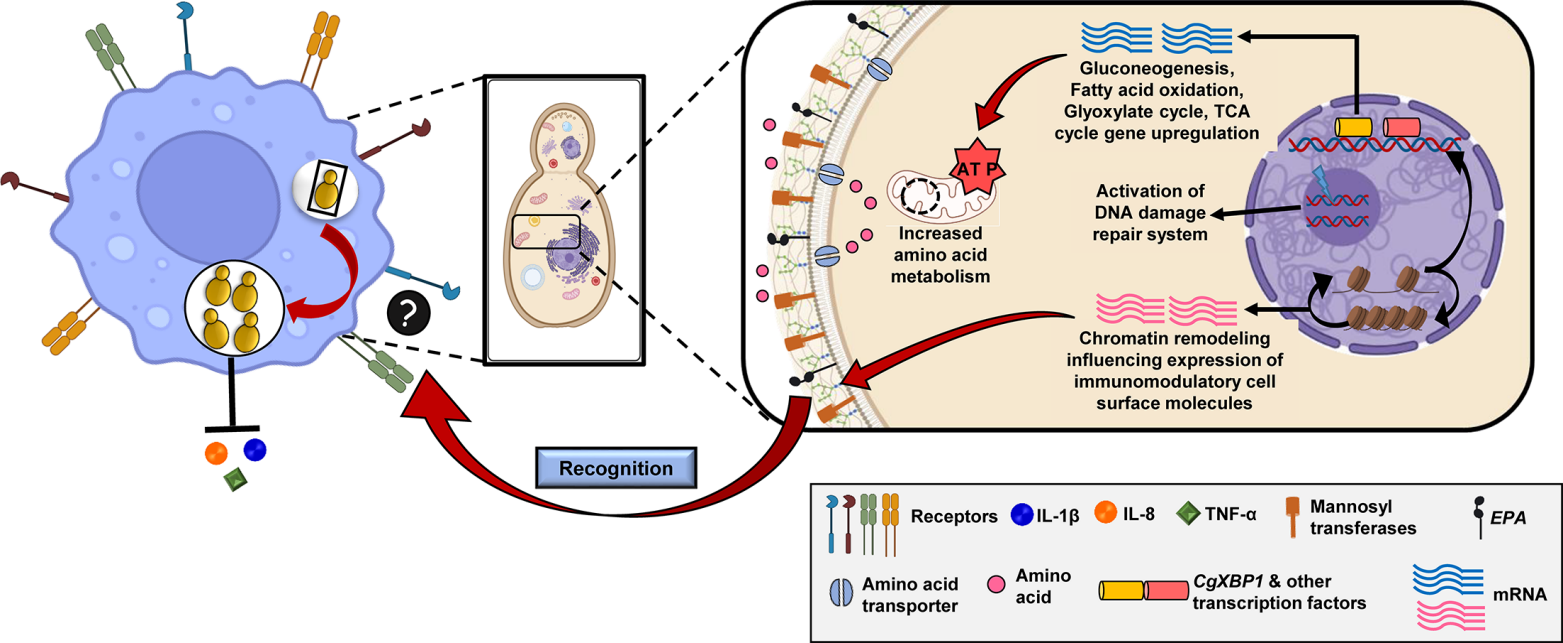
Pictorial depiction of key strategies that *C. glabrata* employs to proliferate in host macrophages. Chromatin remodelling and metabolic reprogramming are pivotal to intracellular replication of *C. glabrata*.

Altogether, reconfiguration of the chromatin, cell surface and metabolism promotes *C. glabrata* proliferation in macrophages by avoiding host recognition, impeding phagosome maturation and facilitating nutrient transport (Fig. 2). Notably, the macrophage-internalized *C. glabrata* have also recently been reported to act as a reservoir of drug-resistant *C. glabrata* infections [41], thereby underscoring the importance of *C. glabrata*-macrophage dynamics for the antifungal therapy against *C. glabrata* infections.

### *C. glabrata*–neutrophil dynamics

Neutrophils, the highly abundant granulocytes in the blood, contain multi-lobed nuclei and are a vital component of the innate immune system [42]. Earlier studies have reported an infiltration of mononuclear cells in *C. glabrata*-infected host tissues [43,45], suggesting that neutrophil chemotaxis may play a less prominent role in the host immune response against *C. glabrata* infections. Further, in a human whole blood infection model, *C. glabrata* was not taken up by neutrophils, despite coming in close vicinity, while it was rapidly engulfed by monocytes, with *C. glabrata* largely inducing a monocytic response, in contrast to the neutrophil-focussed response of *C. albicans* [45]. In spite of this, host neutrophils are capable of phagocytosing and killing *C. glabrata* through reactive oxygen species generation, and neutrophil extracellular trap (NET) formation and release, and releasing killed *C. glabrata* that perhaps aids in activating the subsequent adaptive immune response [26,46,49]. Importantly, in a rat venous catheter model, neutrophils released NETs in response to *C. glabrata* infections [48]. Further, *C. glabrata* challenge led to reduced reactive oxygen species generation in neutrophils of the Dectin-2^−/−^ mice, thereby implicating Dectin-2 receptor in neutrophil-mediated control of *C. glabrata* infection [40]. However, Chen *et al*. reported that *C. glabrata* challenge invoked a similar reactive oxygen species production response in Dectin-2-deficient and wild-type neutrophils; this discrepancy could partly be due to different experimental methodologies employed including varied multiplicity of infection [29]. Notably, Dectin-1-deficient neutrophils were compromised in their ability to kill *C. glabrata* due to impaired reactive oxygen species induction [29]. Moreover, the SKAP2 adapter protein has been shown to be required for *C. glabrata*-stimulated reactive oxygen species production and the consequent neutrophil-mediated killing of *C. glabrata* [50]. Similarly, in neutrophils, the spleen tyrosine kinase, which is activated through lectin-binding receptor signalling, has been reported to be essential for NET formation, TNF-*α* secretion, reactive oxygen species production and *C. glabrata* clearance [49]. Of note, although neutrophils efficiently employ their arsenal of phagocytosis, oxidative burst and extracellular trap formation to capture and kill *C. glabrata*, the traits of biofilm formation, 3′-nucleotidase/nuclease activity, robust reactive oxygen species detoxification systems and IL-8 secretion suppression aid *C. glabrata* in escaping neutrophil-mediated clearance [48,51,53].

Importantly, the transcriptional reprogramming of *C. glabrata* cells, upon neutrophil phagocytosis, included activation of the genes involved in lysine and methionine metabolism, gluconeogenesis, trehalose utilization, glyoxylate cycle, sterol, amino acid and ammonium transport and oxidative stress response processes, along with CgYapsin and autophagy genes [46]. Contrarily, genes implicated in the cell wall mannan biosynthesis, *β*-glucan assembly, sugar transport and synthesis of proteins, ribosomes and ergosterol were downregulated in *C. glabrata* in response to the neutrophil milieu [46]. Of note, the physiological relevance of these differentially expressed genes for *C. glabrata* survival in neutrophils is yet to be fully determined, though peroxiredoxin-encoding *CgTSA1* and *CgTSA2* genes are required for *C. glabrata* survival in neutrophils [51], and genes coding for CgYapsins (*CgYPS-11*), *α*-mannosyltransferases (*CgANP1* and *CgMNN2*) and *β*-mannosyltransferases (*CgBMT2-6*), glyoxylate cycle enzymes (*CgICL1* and *CgMLS1*), trehalase enzymes (*CgATH1*, *CgNTH1* and *CgNTH2*), oxidative stress response transcriptional factor (*CgSKN7*) and the cell cycle and cytokinesis-regulating transcriptional factor (*CgACE2*) modulate *C. glabrata* fitness and/or virulence in the mouse systemic candidiasis, gastrointestinal colonization, urinary tract infection or colitis models [18,22, 54,59]. Overall, further studies are warranted to precisely decipher the role that neutrophils play in controlling *C. glabrata* infections, as well as the *C. glabrata* proteins that protect it from the host neutrophil-mediated killing and elimination.

### *C. glabrata*–epithelial cell interplay

*C. glabrata* is a commensal of host mucosal surfaces and adhesion to the host tissue is pivotal to its colonization potential [1,2]. *C. glabrata* utilizes glycosylphosphatidylinositol-anchored cell surface adhesins, encoded by multigene families, to attach to host tissues including oral, ovary, stomach, vaginal and kidney epithelial cells, with epithelial cells at the mucosal surfaces being the primary barrier to infections [3,34, 35, 53]. However, *C. glabrata* has neither been reported to invade into epithelial cells *in vitro* nor degrade the adherens junction protein, E-cadherin [60,61]. Similar to interactions with macrophages, *C. glabrata* infection of epithelial cells causes a very low level of damage to host epithelial cell model systems *in vitro* [3]. The TLR-2 receptor in the rat tracheal epithelial cells has been implicated in *C. glabrata* recognition which resulted in NFκB activation and TNF-*α* and IL-6 secretion [62]. Similarly, the activation of NFκB, via lactosylceramide receptor CDw17 signalling in the oral epithelial cells and phosphorylation of the *β*-glucan-binding ephrin type-A receptor 2 (EphA2) in the human oral keratinocytes, has been reported in response to *C. glabrata* infections [63,64].

Two recent studies have highlighted the role of the host actin cytoskeleton in *C. glabrata*–epithelial cell interplay. Castillo-Cruz *et al*. have reported that *C. glabrata* promotes actin and microtubule rearrangements in A549 human lung epithelial cells to facilitate its uptake, followed by blocking of endosome maturation and downregulation of autophagy induction to persist and replicate in epithelial cells [65]. Patra and Kaur have demonstrated an essential role for the CgYapsin family of aspartyl proteases in suppression of the proinflammatory cytokine (IL-6 and IL-8) secretion in A-498 kidney epithelial cells by targeting Arpc1B (a subunit of the actin branching-regulating Arp2/3 complex) protein [53]. Further, the diminished IL-8 secretion disrupted epithelial cell-neutrophil communication, leading to reduced infiltration of neutrophils towards *C. glabrata* and enhanced *C. glabrata* survival [53].

Of note, the human albumin protein has been shown to substantially enhance the damage potential of *C. glabrata* to vaginal epithelial cells [66]. Similarly, *C. glabrata* cell wall proteins, Epa8, Epa19, CAGL0F00181p, Awp2 and Awp7, have been implicated in its adhesion to *C. albicans* hyphae, which aids in *C. glabrata* oral colonization in an oropharyngeal candidiasis model [67]. Further, the host mitochondrial processes and type 1 IFN response were activated in the vaginal epithelial cells in response to *C. glabrata* infections [68]. In this context, it is worth noting that *C. glabrata*-invoked type I IFN responses have been reported to dysregulate host zinc and iron homeostasis and help *C. glabrata* evade and persist in the host [69,70]. Recently, a unique *C. glabrata* secretory protein CgYhi1, containing a functional pentapeptide motif, has been reported to induce hyphae formation in *C. albicans* during co-culturing, with filamentation triggering requiring no physical contact between the two *Candida* species [71], thereby raising the possibility that *C. glabrata* may secrete such proteins to damage the host tissue indirectly.

Although the above-mentioned studies shed light on the core of the *C. glabrata*–epithelial cell crosstalk, more in-depth studies are needed to decipher how host actin cytoskeletal changes and type I IFN responses govern the endocytosis and replication of *C. glabrata* in epithelial cells. Additionally, it is imperative to determine how *C. glabrata* modulates the communication between epithelial cells and phagocytic cells to promote its survival in the host.

### Interaction of *C. glabrata* with dendritic cells, natural killer cells and endothelial cells

Dendritic cells serve as a bridge between the innate and the adaptive immune system [15]. *C. glabrata* infection of the bone marrow-derived conventional dendritic cells led to *C. glabrata* phagocytosis, phagolysosome maturation and yeast Pathogen-Associated Molecular Pattern recognition by TLR7 present on the endosomes [72]. *C. glabrata* also stimulated the TLR-specific cytoplasmic MyD88 adaptor activation and phosphorylation of intracellular Src/Syk family kinases, resulting in elevated IFN-*β* secretion by dendritic cells, with type I IFN signalling facilitating *C. glabrata* persistence during systemic infections [72]. Further, the cell wall *β*-glucan in *C. glabrata* has been reported to interact with the complement receptor 3 and the Dectin-1 receptor on the dendritic cells, resulting in induction of the regulatory T cells (Treg) response [73].

*C. glabrata* confrontation has been reported to indirectly activate blood Natural Killer cells and invoke a potent secretion of IL-12 by whole blood and monocyte-derived dendritic cells [74]. Of note, *C. glabrata* is also known to be killed by the mouse Natural Killer cells [32]. Further, *C. glabrata* has been shown to be recognized by Natural Killer cells via the NKp46 (human)/NCR1 (murine) cell surface receptor [32]. This Natural Killer cell recognition, which is mediated by *C. glabrata* cell surface adhesins, Epa1, 6 and 7, is essential to control *C. glabrata* infections, as NCR1^−/−^ mice displayed diminished *C. glabrata* clearance [32].

The capability to invade into host endothelial cells is pivotal to cause disseminated infections. Although *C. glabrata* is not known to possess a high degree of invasiveness, it was able to traverse across the endothelial barrier in a human umbilical vein endothelial cell model system [75]. Additionally, *C. glabrata* invaded the human umbilical vein endothelial cells via a bridging molecule (vitronectin and kininogen, human serum proteins)-dependent endocytosis mechanism [76]. Further studies investigating the *C. glabrata*-endothelial cells interplay have shown that on the *C. glabrata* front, two mannosyltransferase enzymes CgAnp1 (*α*-1,6-mannosyltransferase) and CgMnn2 (*α*-1,2-mannosyltransferase), two cell wall adhesins CgPwp7 and CgAed1, and the silencing protein CgSir3 govern *C. glabrata* adherence to the endothelial cells [22,77, 78]. While on the host front, the *α*-adrenergic 1A, ANG II type I, VCAM-1 and the endothelin-2 lectinic G protein-coupled receptors on the coronary endothelial luminal membrane have been implicated in binding to *C. glabrata* [22,77, 78]. These findings collectively suggest that *C. glabrata* is able to bind as well as invade the host endothelial cells.

Overall, the preceding studies notwithstanding, detailed molecular analyses are required to better understand the capabilities of *C. glabrata* that permit it to circumvent the multiple cellular systems of the host defence pathways.

### Adaptive immune system

T-cell-driven immunity, particularly T helper 1 (Th1) and Th17-type responses, primarily drive the host adaptive immune response against pathogenic fungi and provide protection [79]. However, our understanding of adaptive immune response towards *C. glabrata* infections is limited. *C. glabrata* invoked a surface adhesin CAGL0B00154p-specific intestinal IgA response in mice [80], while systemic *C. glabrata* infections triggered an immunosuppressive response consisting of Treg expansion and IL-10 secretion in mice [73]. *C. glabrata* exposure also led to IL-17A secretion by the primed pALS (*C. albicans* Als1/3 adhesin peptide, recognized by Th cells)-specific T cells [81], and higher IL-10 (Th2 response cytokine) secretion by the human peripheral blood mononuclear cells [82]. Further, the Dectin-2 receptor deficiency not only resulted in diminished *C. glabrata* clearance from the murine kidneys but also decreased the secretion of Th1 and Th17 response-associated cytokines *ex vivo* by restimulated splenocytes [40]. Similarly, Dectin-1 has also been shown to be important for activating Th1 and Th17 responses [29]. Moreover, the mannan component of the *C. glabrata* cell wall led to an increase in CD4^+^CD28^+^ T-cell population as well as triggered dendritic cell maturation via increased expression of the costimulatory molecules CD80 and CD86 [83]. Altogether, these results point towards an important role for the host Th cell responses in the control of *C. glabrata* infections.

Further, it has been shown that *C. glabrata* engages aspartyl proteases to cleave C3 and C3b components and inhibit all three pathways, classical, lectin and alternative, of the complement system that connects adaptive and innate immunity [84]. Moreover, the C3-deficient mice exhibited increased susceptibility to *C. glabrata* systemic infections [85] and C1q binding to *C. glabrata* activated the classical complement pathway independent of the antibodies [84]. Additionally, the binding of *C. glabrata* to complement regulators, factor H, factor H-like protein (FHL-1) and C4b-binding protein (C4BP) [86,87], could aid in evading the complement-mediated host immune response.

### *C. glabrata* persistence and dissemination in animal models

In addition to the aforementioned *in vitro* cell culture model systems, many whole animal model systems including both invertebrate (*G. mellonella*, *Drosophila melanogaster* and *Caenorhabditis elegans*) and vertebrate animals (mouse models of systemic candidiasis, oropharyngeal candidiasis, gastrointestinal candidiasis, vaginal candidiasis, gut-disseminated candidaemia, haematogenous disseminated candidiasis, dextran sulphate sodium-induced colitis and urinary tract infections and the rat model of denture stomatitis) are being used to investigate various aspects of *C. glabrata* pathogenesis [3]. In these host model systems, the host inflammatory response generation, host tissue destruction, host viability, and *C. glabrata* survival, persistence, replication and dissemination within the host body are the primary readouts of *C. glabrata* pathogenicity, reviewed in detail by Askari and Kaur [3]. Altogether, evidence from both *in vitro* and *in vivo* model systems supports the hypothesis that *C. glabrata* does not provoke a strong inflammatory response.

Furthermore, *C. glabrata* has been shown to be disseminated to multiple organs including the kidneys and the brain in the mouse model of systemic candidiasis (intravenous route of infection) and to the liver and kidneys in the mouse model of dextran sulphate sodium-induced colitis (oral route of infection) [28,88]. Notably, the extraintestinal dissemination of *C. glabrata*, despite caecum colonization, was not observed in the antibiotic-treated mouse model of gastrointestinal candidiasis (oral route of infection), while *C. glabrata* was found to disseminate to liver and kidneys in the antibiotics and cyclophosphamide-treated murine model of gut-disseminated invasive candidiasis (intragastric route of infection) [89,90]. Further, *C. glabrata* has also been reported to persist within the peritoneal cavity in a mouse model of intra-abdominal candidiasis (intraperitoneal route of infection) [91]. While reflecting the distinct behaviour of *C. glabrata* in varied infection models, these studies also collectively highlight the major gaps in our understanding of primary commensalism and pathogenicity drivers of *C. glabrata*. Since the human gut appears to be the primary endogenous source of bloodstream infections in patients [2], it is imperative to decipher how *C. glabrata*, in spite of possessing low invasion potential, is able to cross the epithelial barrier to reach the bloodstream as well as disseminate to multiple organs in the host during invasive infections. As the epithelial cell shield is usually weakened upon medical device insertion, injury, burns, surgery, bacterial overgrowth owing to antibiotic usage or invasive fungi-induced damage, it is possible that this non-intact epithelial cell barrier facilitates *C. glabrata* dissemination to the blood and other organs. A systematic analysis testing these possibilities will be immensely useful.

Of note, despite the wide usage of animal models to study *C. glabrata* pathogenesis, their clinical relevance could be limited to a certain extent, as these models neither mimic the human patient-associated conditions including microbiota, insertion of medical devices, neutropenia/immunosuppression, gut dysbiosis, serious underlying diseases, varied therapeutic regimens (chemotherapy, antiretroviral therapy etc.) and human genetics and diversity, nor reproduce all facets of human infections, in entirety. These limitations notwithstanding, the whole animal models are useful in testing new antifungal drugs in controlled settings in ways that are not possible in patients, integrating pharmacokinetics, safety, drug interactions, pharmacodynamics and efficacy *in vivo* [92,93]. Additionally, the animal models have been instrumental to identify the immune receptors and signalling pathways that evoke antifungal immunity and provide protection against *C. glabrata* infections [3,29, 40, 89, 94]. The selection of appropriate infection site, drug dosage, drug delivery route, treatment timings (prophylactic, empiric and therapeutic) and usage of immunocompetent and immunosuppressed animals, transgenic and old diabetic animals and clinical *C. glabrata* isolates, along with studying the effect on disease progression stages, will help minimize the gap between animal testing and clinical success.

### *C. glabrata*–host microbiota interplay

*C. glabrata*, a part of the human gut microflora, probably intimately interacts with the gut bacterial residents and aids in gastrointestinal tract homeostasis, with a decrease in the anaerobic bacterial population resulting in *C. glabrata* overgrowth in a mouse dextran sulphate sodium-induced colitis model and inflammatory bowel disease patients showing *C. glabrata* overabundance [88,95, 96]. Recently, the presence of *Enterococcus faecalis* and gut anaerobes has been shown to be associated with increased *C. glabrata* organ dissemination in a mouse candidiasis model, suggesting that the gut dysbiosis may contribute to *C. glabrata* translocation and infections [97]. Further, in the vaginal niche, *C. glabrata* co-exists with *Lactobacillus* spp., and the mitogen-activated protein kinase of the high osmolarity glycerol pathway, CgHog1, protected *C. glabrata* from high lactic acid levels and low pH stress as well as against the vaginal *Lactobacillus species* in co-culture experiments [98], indicating that CgHog1 may act as a key determinant of *C. glabrata* commensalism in the vaginal microbiome. Notably, *Limosilactobacillus fermentum*, an inhabitant of the human gut and vaginal tract that metabolizes cholesterol, inhibited *C. glabrata* growth via ergosterol depletion in a co-culturing-based growth study [99], raising the possibility of metabolic shifts occurring in *C. glabrata* during co-residence in host settings. Importantly, the cross-kingdom interactions of *C. glabrata* are also reflected in its ability to enhance *Clostridioides* difficile virulence in mice [100]. Altogether, these studies point towards the complex and multifaceted relationships of *C. glabrata* with the host bacterial microbiota; however, more detailed investigations are warranted to decipher the mechanisms by which metabolic competition, impact on immune response generation and pathogenesis attribute expression are governed amongst *C. glabrata* and the bacterial and fungal constituents in varied host niches.

Further, *Candida* species can form single as well as mixed biofilms under clinical settings [2,101, 102], with *C. glabrata* biofilms being proficient in thwarting the neutrophil attack [48]. Notably, an analysis of the *C. glabrata* biofilms, developed on the serum-coated triple-lumen catheters implanted subcutaneously in the back in a rat model, revealed densely packed yeast cells in an extracellular matrix, biofilm persistence up to 9 days and *C. glabrata* cell dissemination into the tissue that surrounded the catheters [103], thereby providing valuable insights into biofilm-associated *C. glabrata* infections. Although the relationship between *C. glabrata* and bacterial biofilms *in vivo* remains largely unexplored, the culture supernatant of *Staphylococcus aureus* triggered apoptotic cell death in *C. glabrata* during *C. glabrata–S.aureus* mixed biofilms *in vitro* [104]. Similarly, *Pseudomonas aeruginosa* inhibited biofilm development in *C. glabrata* [105], and effectors of the type VI secretion system of a gram-negative, opportunistic pathogen *Serratia marcescens* caused *C. glabrata* cell death [106], underscoring the antagonistic relationship between these opportunistic pathogens. Recently, *C. glabrata* and *Klebsiella pneumoniae* have been shown to form mixed biofilms on peripheral venous catheters *in vitro* [107]. Overall, more elaborate studies are required to advance our understanding of single, dual-*Candida* species and polymicrobial biofilm formation under *in vitro*, *in vivo* and in immunocompromised patients to better manage hospital-acquired *C. glabrata* infections.

### *C. glabrata* evolution within the host

*C. glabrata* proficiently adapts within the host environments in part due to its genome plasticity that may arise from aneuploidy, copy number variations and mutations that confer fitness advantages during selective pressures imposed by the antifungal drugs, nutrient scarcity and the host immune system [2,4,7]. Importantly, duplication of genes coding for the cell wall adhesins, reconfiguration of the adhesin-encoding subtelomeric regions and mutations/polymorphisms in adhesin genes or gene mutations enhancing adhesin gene expression are an integral part of genomic adaptation mechanisms in clinical isolates of *C. glabrata* [2,108,113], thereby establishing cell surface adhesins as a key virulence determinant of *C. glabrata*. Of note, Epa1 adhesin of *C. glabrata* has recently been identified as an immunostimulatory molecule, with its downregulation being pivotal to evade the proinflammatory response of host macrophages [31].

The sequenced *C. glabrata* reference strains revealed that the haploid genome of *C. glabrata* is organized into 13 chromosomes and codes for 5272 ORFs [114,116] (http://www.candidagenome.org). *C. glabrata* and its close relative *S. cerevisiae* belong to the whole genome duplication group, with *C. glabrata* undergoing reductive genome evolution [116]. *C. glabrata* has also specifically lost genes belonging to families *SNZ*, *SNO* and *PHO* and *DAL* cluster [117]. Further, the nicotinic acid, biotin, pyridoxine and thiamine auxotrophies of *C. glabrata* are thought to be important for its commensal and pathogenic lifestyles [2,4, 118], with nicotinic acid limitation in urine being associated with derepression of a cell surface adhesin Epa6 and establishment of urinary tract infections in a mouse model [118]. Of note, in light of the biotin auxotrophy, the biotin uptake and regulatory system is important for the intracellular proliferation of *C. glabrata* in host macrophages and persistence during disseminated infections [27]. Moreover, the thiamine auxotrophy that is owing to a partial loss of the thiamine biosynthetic pathway may not be directly linked with *C. glabrata* commensalism/virulence, as other species, which are phylogenetically related to *C. glabrata* but not associated with humans, also exhibited similar thiamine biosynthetic deficiency [117,119]. Of note, *C. glabrata* genome also contains large tandem gene arrays including *MNT3* (code for mannosyltransferases) and *YPS* (encode aspartyl proteases) gene assemblies [108,117]. It will be intriguing to determine the variability and selection of these gene arrays under hospital settings.

Notably, the clinical (serial/non-serial) isolates of *C. glabrata* (recovered from various sites including the blood, stool, abdominal fluid, urine, mouth, vaginal secretion and abscess fluid) have been reported to exhibit genomic arrangements, aneuploidy, spontaneous ploidy switching, small chromosomes, genetic diversity, varied karyotypes, clump-like morphology, genetic recombination, enrichment of non-synonymous changes in cell wall protein-coding genes, copy number variations in adhesin and drug resistance genes, polymorphism in regulatory regions of yapsin-coding genes *CgYPS1* and *CgYPS7*, many retrotransposons, microevolution, hyper-variable mitochondrial genomes and common clonal origin in some drug-resistant isolates [2,108,113, 120]. Further, the whole-genome sequence analysis of 151 global *C. glabrata* isolates has shown a high genetic diversity with a nucleotide diversity of 0.00665, which is 2.2-fold higher than that reported for *C. albicans* [112].

Importantly, independent expansion of the multiple cell wall adhesin-coding gene families is an important virulence signature of *C. glabrata* that probably enables efficient adherence to a wide variety of tissue environments and abiotic surfaces and imparts competence and vigour in the host [2,34, 111, 117]. *C. glabrata* also uniquely possesses huge cell wall adhesin proteins [115]. Additionally, the expansion of *EPA* genes is linked with pathogenicity in the *Nakaseomyces* clade [117], with *EPA* genes also being under positive selection across various sequence types of *C. glabrata* [112]. Further, the evolution and functional diversification of adhesins, belonging to the Epa protein family, have been attributed to the variable structural hot spots in their ligand-binding pockets [128]. Of note, the substantial genetic and phenotypic variations between closely related *C. glabrata* strains point towards rapid evolutionary dynamics as well as high adaptability [111].

Further, despite lacking a sexual cycle, *C. glabrata* does possess mating pathway genes including three mating-type loci (*MTL1*, *MTL2* and *MTL3*) and maintains *a* and *α* mating types, with some evidence of limited mating type switching events being reported in clinical settings [110,111, 129,133]. Importantly, although the expression of the mating-type switching-triggered HO endonuclease was fatal in *C. glabrata*, an efficient non-lethal mating-type switching could be induced, independent of the HO endonuclease, via usage of the CRISPR-Cas9 system [134,135]. Whether and how these switching events in *C. glabrata* provide a facile environment for genetic recombination and/or genome evolution and fast adaptability in hospital settings remains to be investigated.

To summarize, these findings collectively underscore the various variations that occur during infections and probably contribute to *C. glabrata* fitness in the clinical context. Identification of major drivers of these observed genetic/phenotypic/structural changes is crucial for our understanding of *C. glabrata* pathogenicity.

### Research gaps between risk factors and *C. glabrata* pathogenicity studies

*C. glabrata* invasive infections are common in the elderly population and in patients with haematological malignancies or chronic conditions such as diabetes mellitus, with *C. glabrata* also being isolated from hospital surfaces, hands of the health care practitioners and lab coats in a Brazilian tertiary hospital, as well as from the environment [1,3,7, 136]. Therefore, the environmental factors and/or host susceptibility are likely to substantially contribute to the establishment, severity and outcome of invasive *C. glabrata* infections. However, our understanding of these factors is rudimentary, as the majority of *C. glabrata* studies are being conducted *in silos* focussing on one virulence-associated trait at a time and the epidemiological studies are limited. Moreover, the currently employed molecular and immunological approaches and the host model systems also do not adequately integrate the clinical aspects of *C. glabrata* infections. In this regard, it is of utmost importance to leverage advanced sequencing technologies including long-read sequencing and Hi-C to obtain gap-free, high-quality nuclear and mitochondrial genome assemblies of both clinical and environmental isolates to better understand the epidemiology, ecological niche/s, transmission routes, disease spectra, genetic recombination and diversity and genomic changes driving virulence gene expansion, host adaptation and drug resistance in *C. glabrata*. Further, studies modelling the patient metadata, immunological status, antibiotic and antifungal usage, chemotherapy and hospital environments will be useful. Additionally, the development and usage of clinically relevant immunosuppressed animal models and medically relevant surfaces including central venous and urinary catheters and titanium/zirconia dental implant surfaces, for antifungal resistance and biofilm formation studies will be the key to a wholesome understanding of *C. glabrata* pathogenicity. Altogether, deciphering how the interplay between host genetics, immunosuppression and clinical environments drives antifungal resistance acquisition and invasive infections is crucial for an effective management of fatal *C. glabrata* infections.

### Future directions

*C. glabrata* primarily relies on stealth, resilience and immune evasion. Evade and persist are the two major acts of its pathogenesis, which are further compounded by the ever-increasing prevalence of antifungal resistance, and these are likely to contribute to morbidity and mortality associated with *C. glabrata* infections. However, despite the importance of a wholesome understanding of the virulence trait landscape, our knowledge of *C. glabrata*–host interplay is quite limited. For example, the virulence-associated features that predict invasive disease are not known. The factors that fuel *C. glabrata* adaptation, invasion and dissemination are yet to be identified. Similarly, our understanding of how the antibiotic and/or antifungal therapy alters *C. glabrata*–microbiota interactions, which may drive *C. glabrata* virulence, is rudimentary. Moreover, how the host susceptibility factors aid in the progression of *C. glabrata* infections, particularly in view of the mononuclear cell recruitment during intravenous infections, remain elusive. Other understudied areas are the interaction analysis of *C. glabrata* with other microbes including *Candida* species in the clinical context, impact of virulence factors on antifungal drug response and the development of diagnostic tools that can rapidly distinguish colonization from true infections. Research in these areas may aid in patient prioritization, optimal antifungal therapy choices and infection source mapping. Finally, as indicated in the previous section, it is high time to move beyond the traditional experimental approaches to dissect the *C. glabrata*–host crosstalk and employ multipronged integrative research strategies that also combine clinical data, disease trajectories and host genetics and susceptibility, and develop a robust framework, amalgamating immune parameters and virulence signatures, to guide diagnosis, aetiology and successful treatment of *C. glabrata* infections.

## Abbreviations

NET: neutrophil extracellular trap
